# Serial testing of healthcare workers for latent tuberculosis infection and long-term follow up for development of active tuberculosis

**DOI:** 10.1371/journal.pone.0204035

**Published:** 2018-09-20

**Authors:** Youngmok Park, Song Yee Kim, Jeong Wha Kim, Moo Suk Park, Young Sam Kim, Joon Chang, Young Ae Kang

**Affiliations:** 1 Division of Pulmonology, Department of Internal Medicine, Severance Hospital, Yonsei University College of Medicine, Seoul, Republic of Korea; 2 Division of Occupational Health, Yonsei University Health System, Seoul, Republic of Korea; University of Cape Town, SOUTH AFRICA

## Abstract

**Background:**

Healthcare workers (HCWs) are at high risk of tuberculosis (TB) infection due to occupational exposure. It is important to diagnose TB infections in HCWs to prevent nosocomial transmission, particularly among immunocompromised patients.

**Objective:**

The aim of this study was to analyze the rate of tuberculin skin test (TST) conversion and to assess the incidence of active TB after the latent TB infection screenings in high-risk HCWs.

**Methods:**

This retrospective cohort study involved 458 HCWs in TB-related departments between 2009 and 2013. All HCWs underwent a TST and a chest radiograph annually; an interferon-γ release assay (IGRA) was performed on the TST-converted subjects. TST-converted and IGRA-positive HCWs underwent treatment for latent TB infection.

**Results:**

The TST conversion rate was 30.3% from 2009 to 2011 in two years, 7.4% from 2011 to 2012, and 17.4% from 2012 to 2013. Eleven subjects out of 42 TST converters (26%) were IGRA-positive; two of them developed into active pulmonary TB during the follow-up period.

**Conclusions:**

There was significant discordance between TST conversion and IGRA results in high-risk HCWs, and active TB developed only in TST-converted and IGRA-positive HCWs. Therefore, the combined use of TST and IGRA for periodic monitoring of TB infections in high-risk HCWs may be useful.

## Introduction

Healthcare workers (HCWs) are at high risk of *Mycobacterium tuberculosis* infection due to occupational exposure.[1] The likelihood of tuberculosis (TB) infection in HCWs is influenced by the patients they care for, type of occupation, the regional prevalence of TB, and the efficacy of TB infection control programs.[1,2] If HCWs are diagnosed with infectious TB, the impact of nosocomial TB transmission can be considerable because of immunocompromised patients in healthcare systems. Therefore, periodic screenings and preventive treatment for latent TB infection (LTBI) for HCWs at high-risk of TB infection (high-risk HCWs) are recommended.[3–5]

Tuberculin skin tests (TST) are used worldwide to diagnose LTBI, whereas interferon-γ release assay (IGRA), which includes QuantiFERON-TB Gold-in-Tube test (QFT-GIT; Qiagen, Hilden, Germany) and T.SPOT TB test (Oxford Immunotec, Abingdon, UK) are used in some countries according to their national TB programs.[3–5] However, no effective method for periodic screening of LTBI in high-risk HCWs in moderate to high TB burden areas has been developed. IGRA offers a potential method of serial testing to diagnose LTBI in HCWs, and it has better specificity than that of TST in one-time screening.[6,7] On the other hand, using IGRA for serial testing is complicated by lack of data to determine the optimum cut-offs and to interpret the results.[8–11]

South Korea has an intermediate incidence of TB (80/100 000 of the population per year),[12] and Bacillus Calmette–Guerin (BCG) vaccination is mandatory within one month of birth. Although current Korean TB guidelines recommend regular screenings in high-risk HCWs, no proper method has been established.[13]

Using TST and QFT-GIT, serial LTBI screening in high-risk HCWs has been conducted in our institution since 2009. The aim of this study was to analyze the LTBI screening data in high-risk HCWs and to assess the incidence of active pulmonary TB in a tertiary referral hospital in South Korea.

## Materials and methods

### Study subjects and screening protocol

This study was conducted at Severance Hospital (Seoul, Republic of Korea), a tertiary referral hospital with approximately 2400 beds. About 600 culture- or smear-positive pulmonary TB patients are managed annually at this institution.

According to the institution’s policy, all high-risk HCWs were recommended to undergo an annual TB screening from October 2009. High-risk HCWs were defined as those who are working at TB-related departments, such as medical intensive care unit, respiratory department of ward and outpatient clinic, emergency department, microbiology laboratory, and radiology department. Each subject underwent a TST, a simple chest radiography, and a medical interview of comorbidities, previous TB history, work duration, and occupational category (i.e., physician, nurse, health aide, technician, and others).

**Fig 1:**
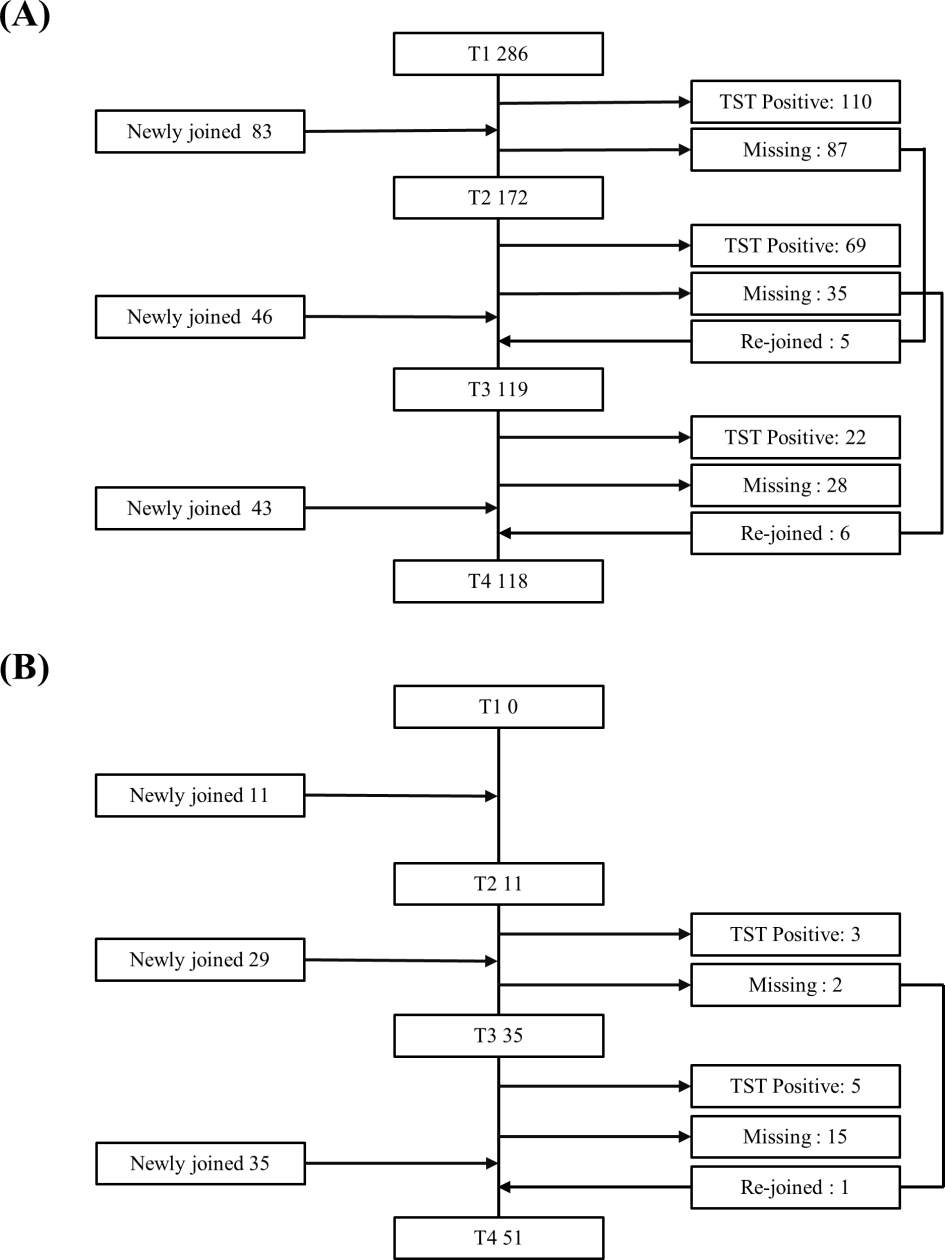
**Study population of (A) total HCWs and (B) HCWs with two-step baseline TSTs.** HCWs, healthcare workers; TST, tuberculin skin test; T1, annual screening in 2009; T2, in 2011; T3, in 2012; T4, in 2013.

We analyzed the screening data of high-risk HCWs who underwent TST at least once between 2009 and 2013. Four TSTs were performed during the study period because only chest radiograph was conducted in 2010. In 2009 (T1), 286 high-risk HCWs participated in the screening. In 2011 (T2), 2012 (T3), and 2013 (T4), 83, 46, and 43 additional high-risk HCWs newly joined the program, respectively, because of movement from other departments or new recruitment. (Fig 1A)

QFT-GIT was tested for the TST-converted subjects. Those who showed TST-converted and QFT-GIT-positive results were strongly recommended to take treatment for latent TB infection with either 3 months of isoniazid and rifampicin, 4 months of rifampicin, or 9 months of isoniazid. High-risk HCWs were followed for the development of active pulmonary TB after 2013 based on the medical records and the results of regular medical exam for all employees.

The study protocol was reviewed and approved by the Institutional Review Board of Severance Hospital, and informed consent was waived by the committee. (4-2017-1055)

### TST and IGRA

A TST was performed on the forearm in accordance with the Mantoux method using a 0.1mL of 2 TU of purified protein derivative RT 23 (Statens Serum Institute, Copenhagen, Denmark). The transverse diameter of the induration was measured in millimeters 48 to 72 hours later. A positive TST was defined as an induration ≥ 10 mm in diameter. TST conversion was defined as a baseline TST induration < 10 mm and a follow-up TST induration ≥ 10 mm with an increment of ≥ 6 mm relative to baseline.[13] Follow-up TST was repeated for subjects with previous negative TST. In addition, for the all new employees from October2011, two-step baseline TSTs were carried out as a medical check-up at the time of employment.

QFT-GIT was performed according to the manufacturer’s instructions. A positive QFT-GIT result was defined as the interferon-γ response of TB antigen minus the negative control of ≥ 0.35 IU/mL and 25% of the negative control value.

### Statistical analysis

Data are presented as numbers (percentage) or medians (range or interquartile range, IQR). Pearson’s chi-squared test or Fisher’s exact test was used to comparing categorical variables, and the Mann-Whitney U-test was used to comparing continuous variables. We used SPSS (v. 18.0; SPSS Inc., Chicago, IL, USA) in data analyzing. In all analyses, P < 0.05 (two-tailed) was taken to indicate statistical significance.

## Results

### Population characteristics

A total of 458 high-risk HCWs were screened at least once by chest radiograph and TST during the study period. The number of subjects who underwent TST screening was 286 in 2009 (T1), 172 in 2011 (T2), 119 in 2012 (T3), and 118 in 2013 (T4). A total of 695 TSTs were performed. (Fig 1A) Also, there were 75 HCWs with two-step baseline TSTs on employment, and 97 TSTs were performed. (Fig 1B)

The baseline characteristics of the 458 participants are presented in Table 1. The median age of the subjects was 29 years (range 22–61 years), and 76.6% were females. Of the subjects, 55.5% were nurses, and the median duration of employment was 46.7 months. Six participants (1.3%) reported a history of anti-TB treatment and 10 (2.2%) had abnormal findings on chest radiographs suggestive of healed pulmonary TB.

**Table 1:** Baseline characteristics of the study population.

		All participants (N = 458)
Age (year), median (range)		29.1 (22–61)
20–29		237 (51.7)
30–39		131 (28.6)
40–49		60 (13.1)
50–59		29 (6.3)
≥60		1 (0.2)
Gender; Female		351 (76.6)
BMI (kg/m^2^), median (range)		20.8 (15.6–34.9)
Smoking status		
Never smoker	423 (92.4)	
Ex-smoker (quit ≥ 1 year)	17 (3.7)	
Current smoker	18 (3.9)	
Healthcare professions		
Physicians		54 (11.8)
Nurses		254 (55.5)
Health aids		46 (10.0)
Laboratory staff		34 (7.4)
Radiology staff		63 (13.8)
Others		7 (1.5)
Work duration (month), median (IQR)		46.7 (18.3–178.3)
<6 month		18 (3.9)
6–24 month		124 (27.1)
24–60 month		104 (22.7)
>60 month		202 (44.1)
Work department		
Outpatient		59 (12.9)
Emergency		126 (27.5)
Inpatient		166 (36.2)
Laboratory/Radiology		104 (22.7)
Comorbidities		
Hypertension		16 (3.5)
Diabetes mellitus		8 (1.7)
Previously TB history		5 (1.1)
Findings on chest X-ray		
Normal		448 (97.8)
Previously healed TB		10 (2.2)
Active TB		0 (0)

Data are presented as numbers (percentages) unless otherwise indicated. Abbreviations: BMI, body mass index; TB, tuberculosis; IQR, interquartile range

### TST results and conversion rates

Table 2 shows the summary of the screening results. The proportion of positive TST results was 38.5% in 2009, 40.1% in 2011, 18.5% in 2012, and 22.9% in 2013. The TST conversion rate was 30.3% from 2009 to 2011 in two years (15.2% per year), 7.4% from 2011 to 2012, and 17.4% from 2012 to 2013.

**Table 2:** Summary of the screening results.

**Total HCWs** **(N = 458)**	N	TST+	%		N	TST Converter	%
T1 (2009)	286	110	38.5				
T2 (2011)	172	69	40.1	T1→T2	89	27	30.3
T3 (2012)	119	22	18.5	T2→T3	68	5	7.4
T4 (2013)	118	27	22.9	T3→T4	69	12	17.4
**HCWs with two-step** **baseline TSTs (N = 75)**	N	TST+	%		N	TST Converter	%
T2 (2011)	11	3	27.3				
T3 (2012)	35	5	14.3	T2→T3	6	1	16.7
T4 (2013)	51	10	19.6	T3→T4	15	3	20.0

Data are presented as numbers. Abbreviations: HCWs, healthcare workers; TST, tuberculin skin test; T1, annual screening in 2009; T2, in 2011; T3, in 2012; T4, in 2013.

For those who had negative results of two-step baseline TSTs from medical exam of recruitment (N = 75), the proportion of positive TST was 27.3% in 2011, 14.3% in 2012, 19.6% in 2013. The TST conversion rate was 16.7% from 2011 to 2012, and 20.0% from 2012 to 2013.

**Fig 2:**
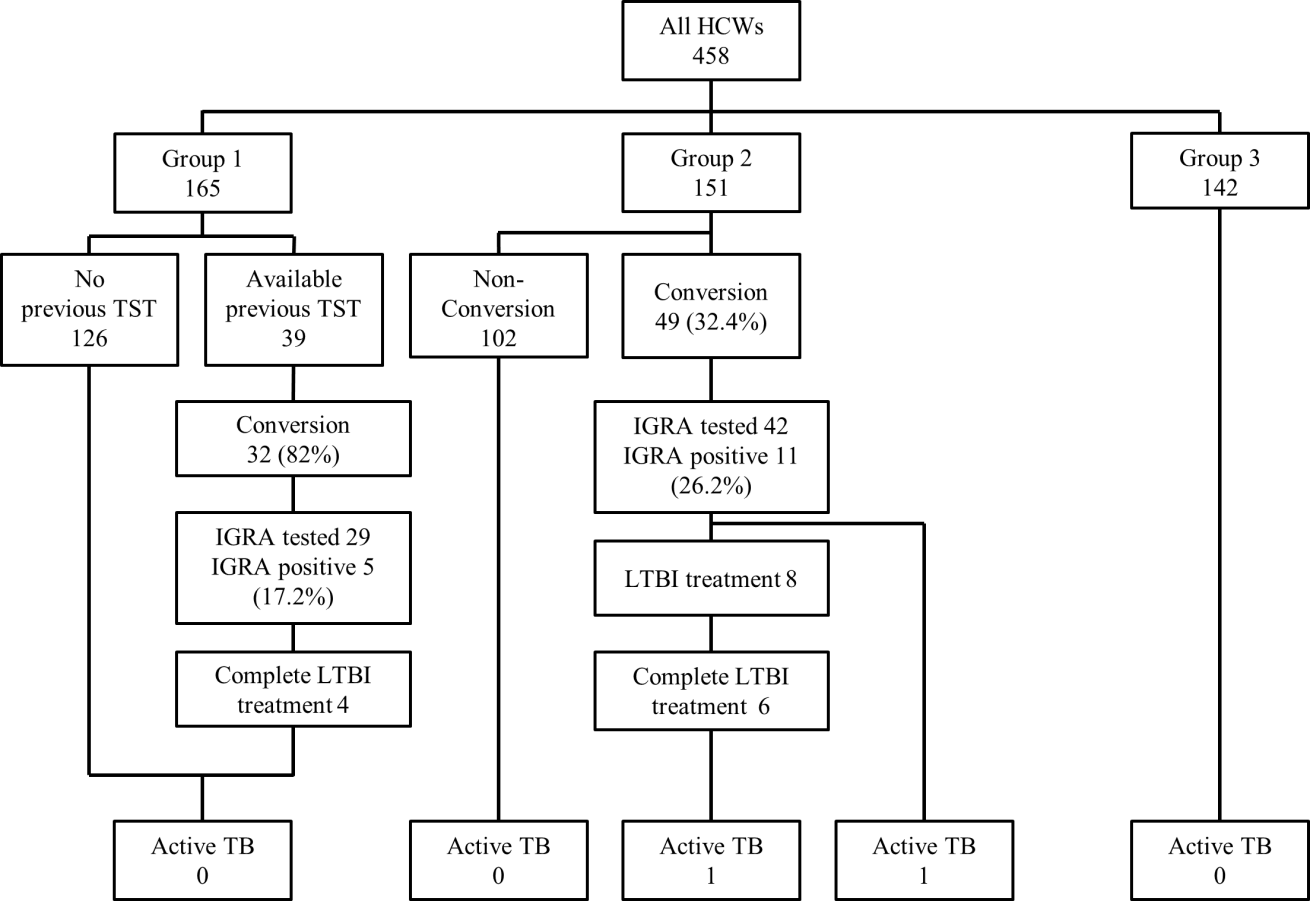
Follow-up for treatment of LTBI and development of active TB. Group 1 comprised HCWs with a positive TST result at their first annual screening. Group 2 comprised HCWs who underwent at least twice TSTs at the annual TB screening. Group 3 comprised HCWs with a negative TST result at their first annual screening but who did not undergo follow-up TSTs. HCWs, healthcare workers; TST, tuberculin skin test; IGRA, interferon-γ release assay; LTBI, latent tuberculosis infection; TB, tuberculosis.

Subjects were classified into three groups according to their TST result at the first screening and the number of TSTs performed during follow-up. (Fig 2) Group 1 comprised 165 HCWs with a positive TST result at the first screening; thus, these subjects did not undergo any further TSTs. Among them, 39 HCWs had prior TST obtained from outside medical records, 32 of which were previously negative (82% TST-converters). Out of 29 who underwent the QFT-GIT, 5 (17.2%) HCWs showed positive results.

Group 2 comprised 151 HCWs who underwent at least two TSTs at the annual TB screening. Among them, 49 (32.4%) HCWs exhibited TST conversion during the study period. TST-converted subjects were older and had a longer duration of employment in the hospital compared to the non-converters. (Table 3) The QFT-GIT was performed in 42 of the TST-converted subjects, of whom 11 (26.2%) showed positive QFT-GIT results. There were no significant differences in characteristics between the QFT-GIT-positive and -negative groups.

**Table 3:** Comparison between converted and non-converted subjects of TST in Group 2.

	All (N = 151)	Non-conversion (N = 102)	Conversion (N = 49)	P-value
Age (year), median (range)	27.5 (22–61)	25.7 (22–61)	30.5 (22–56)	0.002^a^
20–29	87 (57.6)	67 (65.7)	20 (40.8)	
30–39	38 (25.2)	17 (16.7)	21 (42.9)	
40–49	18 (11.9)	15 (14.7)	3 (6.1)	
50–59	7 (4.6)	2 (2.0)	5 (10.2)	
≥60	1 (0.7)	1 (1.0)	0 (0.0)	
Gender; Female	131 (86.8)	91 (89.2)	40 (81.6)	0.190^b^
BMI (kg/m^2^), median (range)	20.1 (16.4–30.5)	20.0 (16.4–27.2)	20.2 (16.6–30.5)	0.403^a^
Smoking status				0.660^b^
Never smoker	140 (92.7)	94 (92.2)	46 (93.9)	
Ex-smoker (quit ≥1 year)	5 (3.3)	3 (2.9)	2 (4.1)	
Current smoker	6 (4.0)	5 (4.9)	1 (2.0)	
Healthcare professions				0.470^b^
Physicians	10 (6.6)	4 (3.9)	6 (12.2)	
Nurses	103 (68.2)	71 (69.6)	32 (65.3)	
Health aides	7 (4.6)	5 (4.9)	2 (4.1)	
Laboratory staffs	13 (8.6)	9 (8.8)	4 (8.2)	
Radiology staffs	16 (10.6)	11 (10.8)	5 (10.2)	
Others	2 (1.3)	2 (2.0)	0 (0.0)	
Work duration (month), median (IQR)	69.1 (36.4–196.1)	57.5 (32.1–179.8)	87.9 (49.5–206.4)	0.034^a^
<6 month	0 (0)	0 (0)	0 (0)	
6–24 month	18 (11.9)	15 (14.7)	3 (6.1)	
24–60 month	49 (32.5)	38 (37.3)	11 (22.4)	
>60 month	84 (55.6)	49 (48.0)	35 (71.4)	
Work department				<0.001^b^
Outpatient	15 (9.9)	4 (3.9)	11 (22.4)	
Emergency	46 (30.5)	25 (24.5)	21 (42.9)	
Inpatient	62 (41.1)	52 (51.0)	10 (20.4)	
Laboratory/Radiology	28 (18.5)	21 (20.6)	7 (14.3)	
Comorbidities				
Hypertension	4 (2.6)	1 (1.0)	3 (6.1)	1.000^b^
Diabetes mellitus	2 (1.3)	2 (2.0)	0 (0)	1.000^b^
Baseline TST induration (mm), median (range)	0 (0–9)	0 (0–9)	1 (0–9)	<0.001^a^
TST conversion duration (month), median (range)			25.5 (12.4–52.2)	
Results of QFT-GIT				
Positive			11 (26.2)	
Negative			31 (73.8)	
Interferon- γ concentration (IU/mL), median (IQR)			0.04 (0.01–0.71)	

Data are presented as numbers (percentages) unless otherwise indicated. Abbreviations: BMI, body mass index; IQR, interquartile range; TST, tuberculin skin test; QFT-GIT, QuantiFERON-TB Gold In-Tube.

^a^ P-values were calculated using Mann-Whitney U-test.

^b^ P-values were calculated using Pearson’s chi-squared test or Fisher’s exact test.

Group 3 comprised 142 HCWs with a negative TST result at their first screening, but who did not undergo follow-up TST due to department translocation, resignation, or non-compliance.

### Follow-up

We strongly recommended treatment for LTBI to the TST-converted and QFT-GIT-positive HCWs and symptom screening and chest X-ray screening per 6 months to the TST-converted and QFT-GIT-negative HCWs. In Group 1, four subjects completed the medication (two received isoniazid and rifampicin for 3 months, one received rifampicin for 4 months, and one received isoniazid for 9 months). The median follow-up duration in Group 1 was 104.5 months (IQR 65.3–104.9 months), and no case of active pulmonary TB occurred during the follow-up.

In Group 2, eight participants started the treatment (three received isoniazid and rifampicin for 3 months, four received rifampicin for 4 months, and one received isoniazid for 9 months), and six completed the course. The median follow-up duration in Group 2 was 104.6 months (IQR, 79.3–105.0 months).

Two cases of active pulmonary TB were detected by symptoms in Group 2 during the follow-up period; both are TST-converted and QFT-GIT-positive nurses. One nurse completed the treatment of LTBI with isoniazid and rifampicin for 3 months. Active pulmonary TB developed at 31 months after completion of medication. The isolated *M*. *tuberculosis* was resistant to isoniazid, ethambutol, and streptomycin. The other nurse who did not take the medication for LTBI was diagnosed with active pulmonary TB at 38 months after TST conversion. Isolated *M*. *tuberculosis* was susceptible to all anti-TB medicines.

The median follow-up duration in Group 3 was 56.5 months (IQR 24.4–104.8 months). Based on their medical records and annual medical check-up for all workers in this institution, no case of active TB occurred in this group during the follow-up. (Fig 2)

## Discussion

In this study, the rate of TST conversion in high-risk HCWs of a tertiary referral hospital in South Korea ranged from 7.4% to 17.4% per year. The proportion of positive QFT-GIT results in the TST-converted HCWs was 26.2%. In addition, active pulmonary TB developed only in the TST-converted and QFT-GIT-positive subjects.

Although the current Korean TB guidelines recommend periodic screening for LTBI in high-risk HCWs,[13] data on the annual risk of TB infection in HCWs in South Korea are limited, particularly for high-risk HCWs. According to a previous study, TST and QFT-GIT conversion rates of nurses after one year in the Korean tertiary care hospital were 21.3% and 14.4%, respectively.[14] Another study showed that 5.7% of nurses and physicians exhibited QFT-GIT conversion after one year of work.[15] Because of the differences in subjects’ characteristics and screening methods between the current and the previous studies, we could not directly compare the risk of TB infection. However, since we focused on TB infection in high-risk HCWs, the annual TST conversion rate of 7.4–17.4% was similar to that of newly employed nurses. The annual incidence of TB infection in high-risk HCWs might be higher than that in the general population (with an expected annual risk of infection of 0.23% in 2005), although the data were not adjusted for age or sex.

In our hospital, active pulmonary TB patients are admitted in isolated rooms with negative-pressure ventilation. Any visitors or HCWs entering the rooms must wear proper respirators such as N95 masks. On the other hand, several patients are being diagnosed as active pulmonary TB in general wards and outpatient clinic, where it is not feasible for HCWs to wear proper protectors. In such circumstances, not only HCWs but also other patients can be placed at high risk of TB transmission. In this point of view, we looked into a correlation between an annual number of TB patients in this institution and TST conversion rate in high-risk HCWs, but there was no direct relationship during the study period. The annual reported number of pulmonary TB patients was 667 in 2011, 627 in 2012, and 435 in 2013. The TST conversion rates were 30.3% from 2009 to 2011, 7.4% from 2011 to 2012, and 17.4% from 2012 to 2013. TST conversion rate between 2009 and 2011 seems relatively high; possible reasons might be on the longer interval of two years, and the boosting effect of TST, since two-step baseline TSTs for the newly employed personnel were performed after 2011.

Discordance between TST and QFT-GIT results for LTBI diagnosis has been reported.[16] High TST conversion rate and the low correspondence of QFT-GIT result might be confounded by weak immune responses and technical assay variability of IGRA.[10,17,18] The United States Centers for Disease Control and Prevention suggested that IGRA may be used in place of TSTs,[4] and it is preferred for BCG-vaccinated individuals.[16] However, the results of serial IGRAs in HCWs show marked variability, and the interpretation is hampered by the lack of consensus regarding the definition of conversion.[19,20] Thus, the optimization of the IGRA process for reliable results is necessary.[18] In addition, the delayed boosting reaction of repeated TST could be one explanation of discordance between high TST conversion and low correspondence of QFT-GIT. Previous studies reported the delayed boosting reaction in two-step baseline TST HCWs who were BCG vaccinated.[21,22]

Our protocol used both TST and QFT-GIT to screen for LTBI in high-risk HCWs. During the near 9 years of follow-up, there were two active TB cases among TST-converted and QFT-GIT-positive subjects, but none among TST-converted and QFT-GIT-negative group. To our best knowledge, this is the first long-term follow-up study of active TB development in high-risk HCWs tested periodically for LTBI in moderate to high TB burden areas.

There is still no effective method for LTBI screening in high-risk HCWs with TST-converted and QFT-GIT-negative subjects. We recommend and symptom screening and chest X-ray screening per 6 months to the TST-converted and QFT-GIT-negative HCWs and are planning to begin a serial screening with QFT-GIT in those HCWs. Since the interpretation of the serial IGRA might be somewhat problematic, further research is needed.

This study has several limitations. First, it was conducted in a single institution with a small number of subjects. Second, we could not perform repeated QFT-GITs in all subjects. The substantial within-subject variability associated with QFT-GIT results hampers generalization of comparison between TST conversion and QFT-GIT results. Third, a considerable number of subjects was lost to follow-up, likely due to transfer to another department, pregnancy, or retirement. Fourth, because we performed two-step baseline TSTs for the newly employed HCWs only after 2011, the TST results before that time may include boost effect. Therefore, as mentioned above, the conversion rate from 2009 to 2011 may have been overestimated. Finally, it is not possible to exclude the chance that the two active pulmonary TB cases were caused by exogenous reinfection considering relatively long interval after TST conversion.

In conclusion, the TST conversion rate in high-risk HCWs of a tertiary referral hospital in a country with an intermediate TB burden ranged from 7.4% to 17.4% per year, and less than 30% of the TST-converted subjects showed positive QFT-GIT results. Active pulmonary TB developed only in two TST-converted and QFT-GIT-positive subjects. Therefore, the combined use of both TSTs and IGRAs for periodic monitoring of new TB infections in high-risk HCWs might be useful in areas with an intermediate TB burden in which BCG vaccination is mandatory.
